# Genomic epidemiology sheds light on the emergence and spread of *Mycobacterium bovis* Eu2 Clonal Complex in Portugal

**DOI:** 10.1080/22221751.2023.2253340

**Published:** 2023-09-06

**Authors:** André C. Pereira, Ana C. Reis, Mónica V. Cunha

**Affiliations:** a Centre for Ecology, Evolution and Environmental Changes (cE3c) & CHANGE – Global Change and Sustainability Institute, Faculdade de Ciências, Universidade de Lisboa, Lisboa, Portugal; b Biosystems & Integrative Sciences Institute (BioISI), Faculdade de Ciências, Universidade de Lisboa, Lisboa, Portugal

**Keywords:** *Mycobacterium bovis*, animal tuberculosis, whole-genome sequencing, phylodynamics, phylogeography

## Abstract

Animal tuberculosis (TB) remains a serious concern for animal and human health. *Mycobacterium bovis* circulates in multi-host systems, dominated by the European 2 clonal complex (Eu2) in Iberia. In this work, we use genomic epidemiology to infer the emergence, spread, and spatiotemporal patterns of Eu2 in the official epidemiological risk area of animal TB in Portugal. Phylogenetic analysis of 144 *M. bovis* whole-genome sequences from cattle, wild boar, and red deer, representing the 2002–2021 period, distinguished three Eu2 clades that evolved independently. The major Eu2 clade underwent phylodynamic inferences to estimate the time and location of outbreaks, host transitions, and spatial diffusion as well. The origin of this Eu2 clade was attributed to the red deer population in the Castelo Branco district, near the border with Spain. Most host transitions were intraspecific (80%), while interspecific transmissions between wildlife species (wild boar-red deer), and between wild boar and cattle, were highly supported. Phylogeographic reconstruction evidenced that most transitions (82%) occur within municipalities, highlighting local transmission corridors.

Our study indicates that *M. bovis* continues to spread at the cattle-wildlife interface within the animal TB hotspot area, possibly driven by the foraging behaviour of wild boar near agricultural lands. Red deer seems to be an important driver of TB within wildlife hosts, while the wild boar links the multi-host wildlife community and livestock. This work highlights the value of combining genomic epidemiology with phylodynamic inference to resolve host jumps and spatial patterns of *M. bovis*, providing real-time clues about points of intervention.

## Introduction

Animal tuberculosis (TB) is a zoonotic infection presenting a major socio-economic burden in countries with endemic areas [1]. In South-Western Europe, animal TB is mainly caused by *Mycobacterium bovis* [2–4], a member of the *Mycobacterium tuberculosis* complex (MTBC). In this region, *M. bovis* circulates in multi-host communities including livestock and wildlife. The most epidemiologically relevant species vary from region to region depending on the ecosystem [2–4].

In Portugal, *Bos taurus* (cattle), *Sus scrofa* (wild boar), and *Cervus elaphus* (red deer) are known reservoirs of *M. bovis* [5, 6]*.* The bovine TB eradication and control programme in Portugal is based on European Union regulations and includes live testing of cattle, culling of suspect animals followed by laboratory confirmation via histopathology and bacteriology, herd quarantine, and pre-movement testing [7]. However, the endemization of *M. bovis* in wildlife hampers the success of the eradication programme specifically focused on bovine TB. In 2011, an epidemiological risk area for animal TB in big game (wild ungulates) was officially defined. In this area, an initial examination of hunted animals by officially trained veterinarians who search for TB-compatible lesions became mandatory, followed by laboratory processing of suspect samples. The adequate disposal of viscera and suspect carcasses was also deemed to follow established regulations [8].

Previous large molecular analyses of *M. bovis* populations from Portugal and Spain based on spoligotyping and single nucleotide polymorphism (SNP) typing schemes revealed the dominance of European 2 clonal complex (Eu2) (around 70% of all strains) in the Iberian Peninsula [9]. Nowadays, whole-genome sequencing (WGS) is being increasingly incorporated into official animal TB control programmes to investigate herd outbreaks and epidemiological links [10–16]. Contrary to classical genotyping analyses that explore less than 1% of the genome and may be biased by the independent origin of identical polymorphisms in unrelated isolates (homoplasy), WGS enables multiple genome-wide analyses that explore the map to reference strategies and polymorphic sites (i.e. SNPs), which have residual reversion rates and homoplasy indices [17].

Recently, genomic surveillance was applied in a pilot study (n = 44) in Portugal to cattle, red deer, and wild boar isolates to reconstruct the *M. bovis* population structure and explore epidemiological links between different hosts [16]. Genomic data supported the branching of the *M. bovis* population into five clades, highlighting the key epidemiological roles of red deer and wild boar on TB maintenance, and multiple introduction events in the study area. In the present work, we extend previous genomic analyses to a considerably wider dataset of *M. bovis* isolates for phylogenetic inferences of evolutionary rates and time scales. We restrict analyses to the major clonal complex, the Eu2 (n = 144), to advance knowledge, at a fine-scale, of phylodynamic processes among and between cattle and wildlife populations in the central-eastern TB hotspot region, located at the periphery of the high-prevalence TB core area of the Iberian Peninsula. We first applied phylogenomic analyses to the whole dataset to disclose the structure of the Eu2 population. Genome data revealed a clock-like behaviour of the major clade, which thus underwent Bayesian-based phylodynamic analyses to tentatively date its introduction, establish epidemiological parameters and identify, with higher resolution, host transitions in transmission chains. Understanding the multitude of transmission and evolutionary scenarios in which *M. bovis* circulates in this multi-host system may guide the improvement of surveillance and control measures.

## Materials and methods

### *M. bovis* isolates and whole-genome sequencing

**Figure 1.**
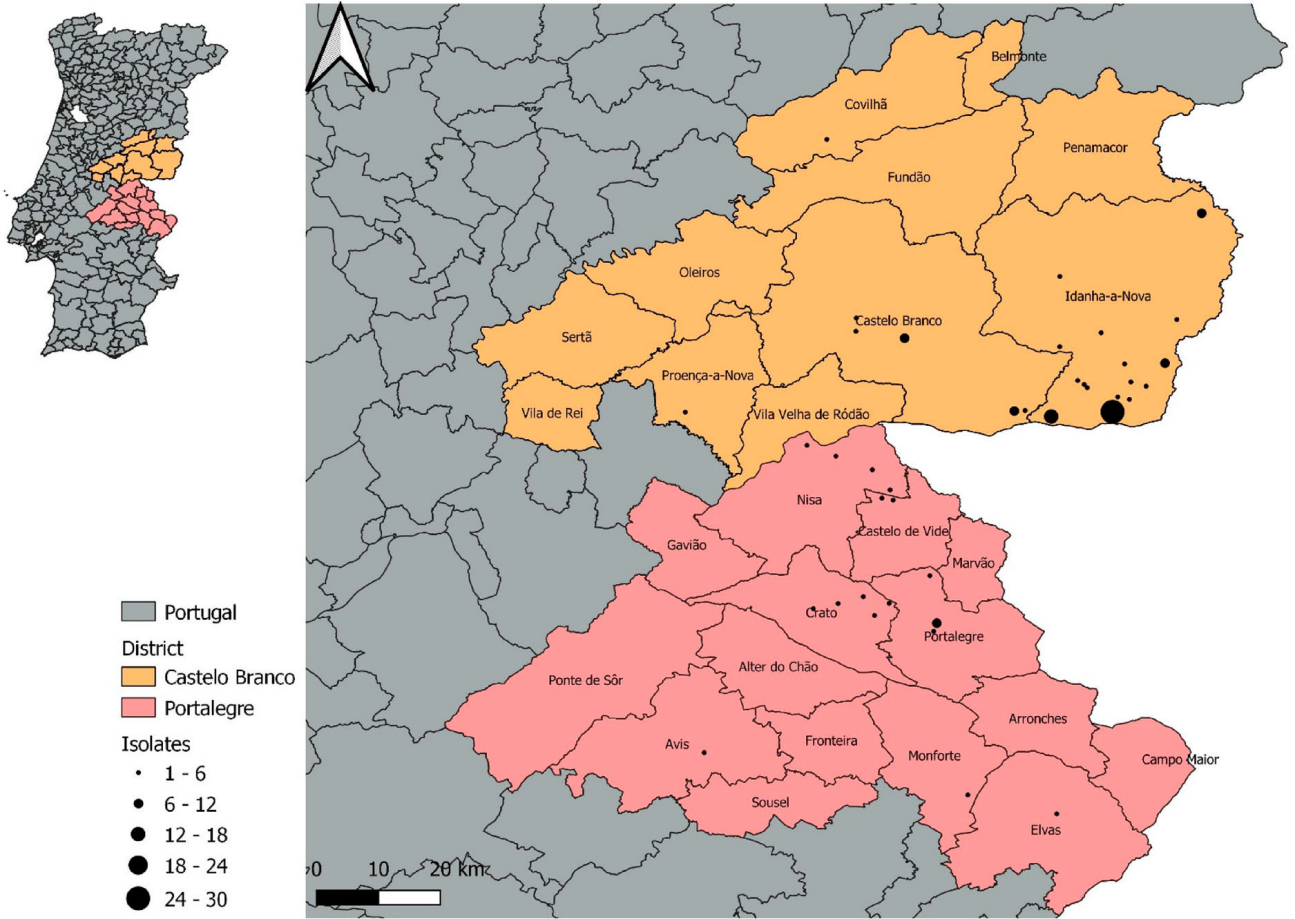
Geographical location of the Eu2 *Mycobacterium bovis* isolates from this study, within the central-east animal TB hotspot area in Portugal.

The 143 *M. bovis* isolates examined in this work were collected between 2002 and 2021 (ranging 19 years) from cattle (n = 45), red deer (n = 51), and wild boar (n = 47) from the central-eastern TB hotspot region in Portugal, located at the periphery of the high-prevalence TB core area of the Iberian Peninsula (Figure 1). This animal TB hotspot comprises the Castelo Branco (n = 107) and Portalegre (n = 36) districts (administrative regions) (Supplementary Table 1).

Sample collection was performed within an official context according to specific national legislation. Cattle tissue samples were obtained after the sanitary slaughter of reactors or from macroscopic TB-like lesions at routine abattoir inspection. Wildlife samples were collected in the epidemiological risk area for TB from hunted animals presenting TB-compatible lesions during veterinarian examination. This dataset was selected for WGS from a wider *M. bovis* dataset using as selection criterium the geographic location from where the isolates were collected (Figure 1).

Sample processing was performed according to the World Organisation for Animal Health (WOAH)’ Manual of Diagnostic Tests and Vaccines for Terrestrial Animals. Cell biomass grown on solid medium (Lowenstein Jensen with pyruvate) or on liquid culture (BD BACTEC™ 9000 or the MGIT™ 960 system) was suspended in phosphate buffer saline (PBS), the cells were harvested by centrifugation and inactivated by heating at 99˚C for 30 min. The supernatant was stored at −20˚C until further use. Isolates were thawed and inoculated in Middlebrook 7H9 (Difco, USA) medium supplemented with 5% sodium pyruvate and 10% ADS enrichment (50 g/L albumin, 20 g/L glucose, 8.5 g/L sodium chloride) at 37˚C in a level 3 biosecurity facility, for 4 weeks. After this period, the culture medium was renewed and regular monitorization was performed until growth was observed.

The genomic DNA of 143 *M. bovis* isolates was sequenced using the Illumina Genome Analyser, according to the manufacturer’s specifications, with the paired-end module attachment. DNA was sequenced by HiSeq (2 × 150 pb) Illumina sequencing technology (Eurofins, Germany). The raw read FASTQ files were used for quality evaluation using FastQC (version 0.11.9), followed by trimming and filtering using Fastp, filtering reads < 75 bp and forcing polyG tail trimming maintaining default parameters [[18], version 0.20.1]. High-quality reads were taxonomically classified using Kraken2 [[19], version 2.1.1], with samples with over 70% of reads belonging to the *Mycobacterium* genus being considered non-contaminated. In case of contamination, reads belonging to the *Mycobacterium* genus were filtered out.

Samples were further analysed using the vSNP pipeline, currently available at https://github.com/USDA-VS/vSNP (accessed on 1 June 2022). Briefly, the pipeline involves a two-step process. Step 1 determines SNP positions called within the sequence, using BWA-MEM to align reads to the reference genome of *M. bovis* AF2122/97 (NCBI accession number LT708304.1). Duplicate reads were removed using the Mark Duplicates tool with Picard (version 2.20.2). SNPs with an allele count (AC) = 2, quality score >150, and map quality >56 were called using FreeBayes (version 1.3.1). The depth of coverage was calculated using Pysam (version 0.18). Uninformative SNPs were excluded. Integrated Genomics Viewer [[20], version 2.9.2] using BAM files was used to validate SNPs and positions with mapping issues or alignment problems. Unreliable positions due to read alignment errors were removed from the analysis, as well as the SNPs that fell within Proline-Glutamate (PE) and Proline-Proline Glutamate (PPE) genes. *In silico* spoligotyping was performed using the vSNP pipeline. The new raw data have been deposited in a public domain server at the NCBI SRA database, under BioProject accession number PRJNA946560 (Supplementary Table 1).

Two whole genome sequences evidenced signs of mixed strains, *i.e.* in which the defining SNPs had ambiguous calls. In these cases, strain separation was performed with SplitStrains [21] using as a reference the genome of *M. bovis* AF2122/27 (LT708304.1), as described in [21] and [22]. Each of these two samples was shown to have two different strains, that were successfully split, originating information corresponding to four different strains. Upon read separation, reads were extracted from the original BAM alignment files and each read set was once again aligned to reference with the vSNP pipeline.

### Phylogenetic analyses

*M. bovis* lineage identification was carried out using KvarQ [[23], version 0.12.2] which assesses lineage-specific SNPs as defined in [24].

Validated SNPs of Eu2 isolates were concatenated, resulting in a single 3661 nucleotide sequence for each isolate. Molecular Evolutionary Genetics Analysis [MEGA-X, [25], version 10.1.8] was used to conduct phylogenetic analysis, using the maximum likelihood method with General Time Reversible (GTR) gamma-distributed with invariant sites, with 4 discrete gamma categories, and 1000 bootstrap inferences. Three isolates from the European 1 (Eu1) clonal complex (BioProject accession number PRJNA946560) recovered from the same study area and period were used as an outgroup to root the tree.

### Microevolution and co-infection analysis

Validated SNPs of the four split strains were concatenated, resulting in a single 560 nucleotide sequence. MEGA-X was used as previously stated. A pairwise SNP distance matrix was also obtained using the number of differences between pairs of isolates.

### Phylodynamic analyses

The temporal signal of the entire phylogenetic tree and all major three clades was explored with TempEst [[26], version 1.5.3] by a root-to-tip test, evaluating the correlation (r^2^) between SNP distance and time. The Least Square Dating (LSD) software [[27], version 0.3-beta] was also run to test through a date-randomization test, with 10 date-randomized datasets, the clock-like behaviour of each clade.

Next, for the clade showing temporal signal, the best-fitting nucleotide substitution model was selected according to the Bayesian information criteria (BIC) model selection implemented by jModeltest2 [[28], version 2.1.10].

After, several Bayesian coalescent Markov Chain Monte Carlo (MCMC) analyses were performed in BEAST2 [[29], version 2.6.2] to select the best set of molecular clock model and demographic prior. Three molecular clock models (strict, relaxed exponential, and relaxed log normal) and three coalescent demographic priors (constant, exponential, and Bayesian skyline population), resulting in nine different models [30–33] were tested. Independent MCMC analyses were run for 100 million generations steps and posterior distributions were sampled every 5000 generations. For all models, two runs were performed, and model parameters were assessed for convergence and satisfactory effective sample sizes (ESS >200) in Tracer [[34], version 1.7.1]. The runs were combined in LogCombiner [[35], version 2.6.6], where trees were also subsampled, and a maximum clade credibility tree was created with median heights (10% burn-in) using TreeAnnotator [[35], version 2.6.3] and visualized in FigTree [[35], version 1.4.4]. Model performance was evaluated by maximum likelihood estimation (MLE) based on path sampling [PS; [36]] and paired comparisons of marginal likelihoods using Bayes Factors [BF; [37]].

Lineages-through-time analysis was performed in Tracer using the best-fitting model to measure strain differentiation during the study period.

### Estimation of epidemiological parameters

Two estimated parameters under the exponential molecular clock assumption (according to the best-fitting model) were obtained: effective population size at present (ePopSize) and the growth rate (growthRate). In the context of infectious diseases, these parameters correspond to ePopSize = Φ / 2 * λ and growthRate = λ – δ, where the Φ is the number of infected hosts at present, the λ is the coalescent rate, and the δ is the rate of becoming uninfectious due to death or recovery (https://github.com/sebastianduchene/tree_model_adequacy/wiki/How-to-use-Tree-Model-Adequacy). These parameters can be used to estimate the effective reproductive number (R_e_), which is the mean number of secondary cases per infected individual, assuming a fully susceptible population: R_e_ = λ / δ. In this study, Φ was assumed according to the stochastic-based estimations of the multi-host community of animal TB in Portugal [38] using the mean inferred value obtained through the analysis of several studies encompassing a time window between 2009 and 2018.

### Host species transmission analyses

A Discrete Ancestral Trait Mapping (DATM) analysis [39, 40] was implemented in BEAST2 using host species as a discrete trait (*i.e.* cattle, red deer, and wild boar), for both symmetric (estimates pathogen transition probability from host species independently of direction) and asymmetric (estimates unidirectional probability for host transition) assumptions, using the best fitting model determined in the previous section. Model performance under both symmetric and asymmetric analyses was compared as previously stated. The relative estimated posterior probabilities (PP) of transition between host species from both symmetric and asymmetric models were obtained.

### Phylogeographic analyses

Phylogeographic inferences were based on DATM analyses using geographic data, such as district (upper administrative level) and municipality (lower level), as discrete traits, under both symmetric and asymmetric assumptions. When municipality information was missing for the major clade, the district’s main municipality was assumed. All phylogeographic models were built using the best-fitting model determined in the previous section. Model performance of all four models was compared by path sampling as previously stated. The relative estimated PP of the transitions and the average diffusion rates between discrete geographical units in both symmetric and asymmetric models were obtained.

The transitions between municipalities inferred by the symmetric model were recovered from internal nodes. Alluvial plots were produced to highlight both intra – and inter-municipality transitions, which were also represented using Cytoscape (version 3.9.1) [41]. Superimposition of the resulting network into a map of the hotspot area of animal TB in Portugal, depicting the mean wildlife density per municipality (using as a proxy the number of wild boar and red deer hunted in those areas between 2000 and 2008 – corresponding to the period in which the majority of inter-municipality transitions occurred), was performed with QGIS (version 3.18.3).

## Results

### Ambiguous SNP calling followed by strain splitting indicates two co-infection cases

**Figure 2.**
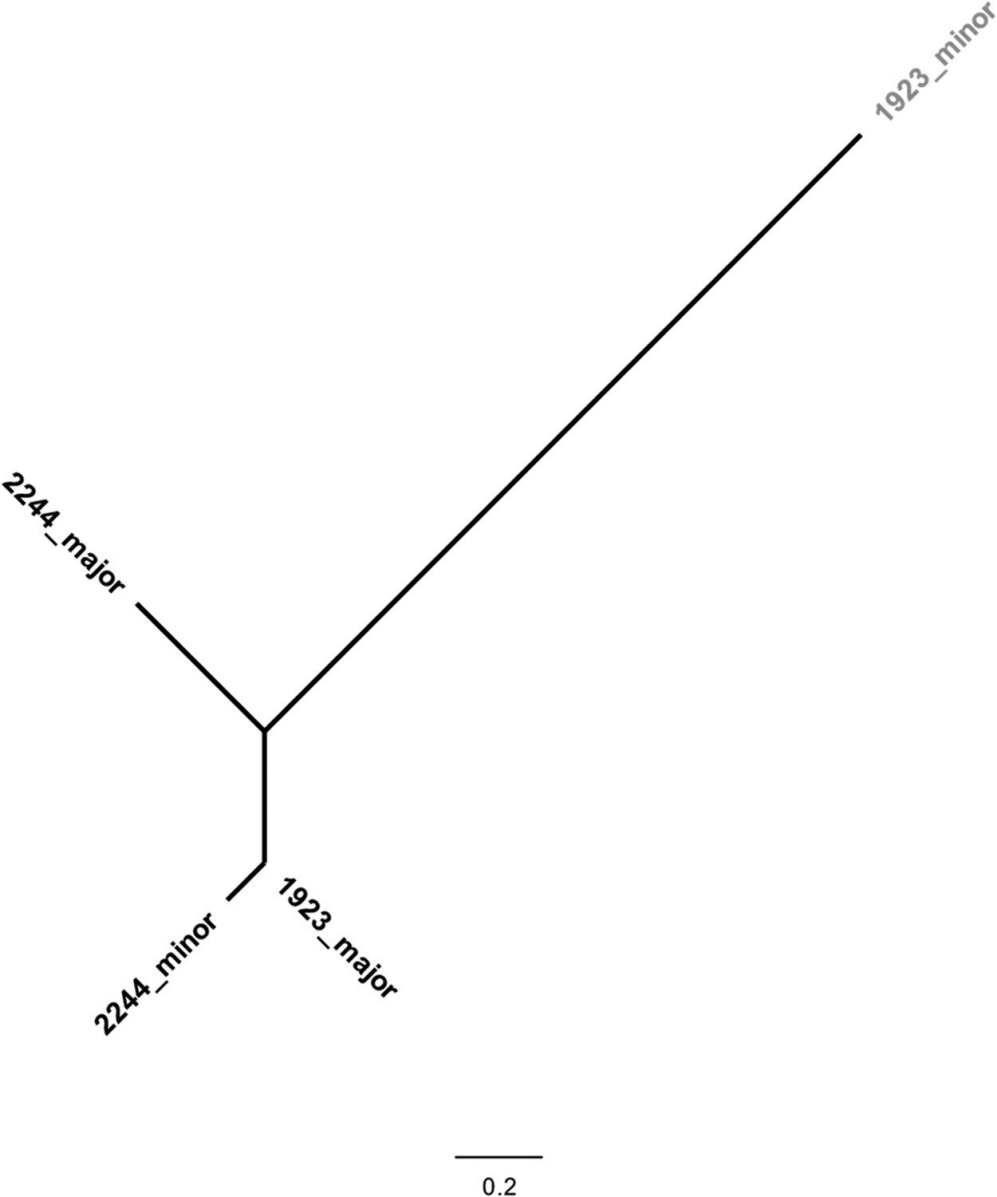
Phylogenetic analysis of coinfecting strains of *Mycobacterium bovis*. A total of 4 *M. bovis* genomes recovered from two different hosts were analysed using a GTR (General Time Reversible) model and maximum-likelihood approach. Major and minor refer to the relative abundance of the strain in the original sample. The scale represents the number of nucleotide substitutions. The strain highlighted in grey belongs to the Eu3 clonal complex, while the remaining belongs to Eu2 clonal complex.

During the analysis of WGS data, two genome sequences (out of 143) were identified by vSNP (https://github.com/USDA-VS/vSNP) as being mixed samples, *i.e.* sequenced DNA extracts in which defining SNPs had ambiguous calls. Those genome sequences were processed using SplitStrains to separate the reads from the different co-occurring strains of *M. bovis*. Each of the two genome sequences corresponded to two different strains, resulting in four recovered strains (Figure 2). For each sample, the recovered strains were identified as major or minor depending on their relative percentage within the sample. These two mixed sample cases were investigated as possible co-infections (>10 SNPs of difference) or intra-host events of microevolution (≤10 SNPs) [17].

Identified mixed samples were isolated from two wild boars (samples 1923 and 2244), with one sample being recovered from Portalegre and one from Castelo Branco (Figure 2). Mixed infections were detected in 2013 and 2015. The two Eu2 strains recovered from sample 2244 differed by 262 SNP (major strain, 95%; minor strain, 5%; Figure 2). In sample 1923, strains differed by a total of 406 SNPs and were identified as Eu2 clonal complex (major strain, 94%) and Eu3 clonal complex (minor strain, 6%; Figure 2). Except for the minor Eu3 strain, which was excluded from further analyses, the filtered read datasets for the split Eu2 major and minor variants were each aligned to reference with the vSNP pipeline and added to the bulk of Eu2 whole genome sequences for further analyses. The entire dataset then became 143 Eu2 genomes plus one from the split 2244 sample.

### Phylogenetic analyses of the Eu2 *M. bovis* population suggest multiple infection sources and transmission pathways

The 144 Eu2 whole genome sequences (i.e. after splitting) presented an average depth of coverage of 261.3X, while the average genome coverage was 99.65% (Supplementary Table 2). Upon SNP identification and validation, the SNP alignment of all 144 genomes yielded a total of 3243 positions.

*In silico* spoligotyping was performed to assess genetic diversity and allow comparison with previous findings. Seventeen spoligotypes were identified, with SB0122 (25%) and SB0121 (24%) being the most prevalent (Supplementary Table 1). SB0122 (31%) was commonly found in the Castelo Branco district, while SB0121 (51%) was dominant in Portalegre. SB0121 was the most frequent in cattle (36%) and wild boar (25%), while SB0122 (35%) was dominant in red deer populations.

**Figure 3.**
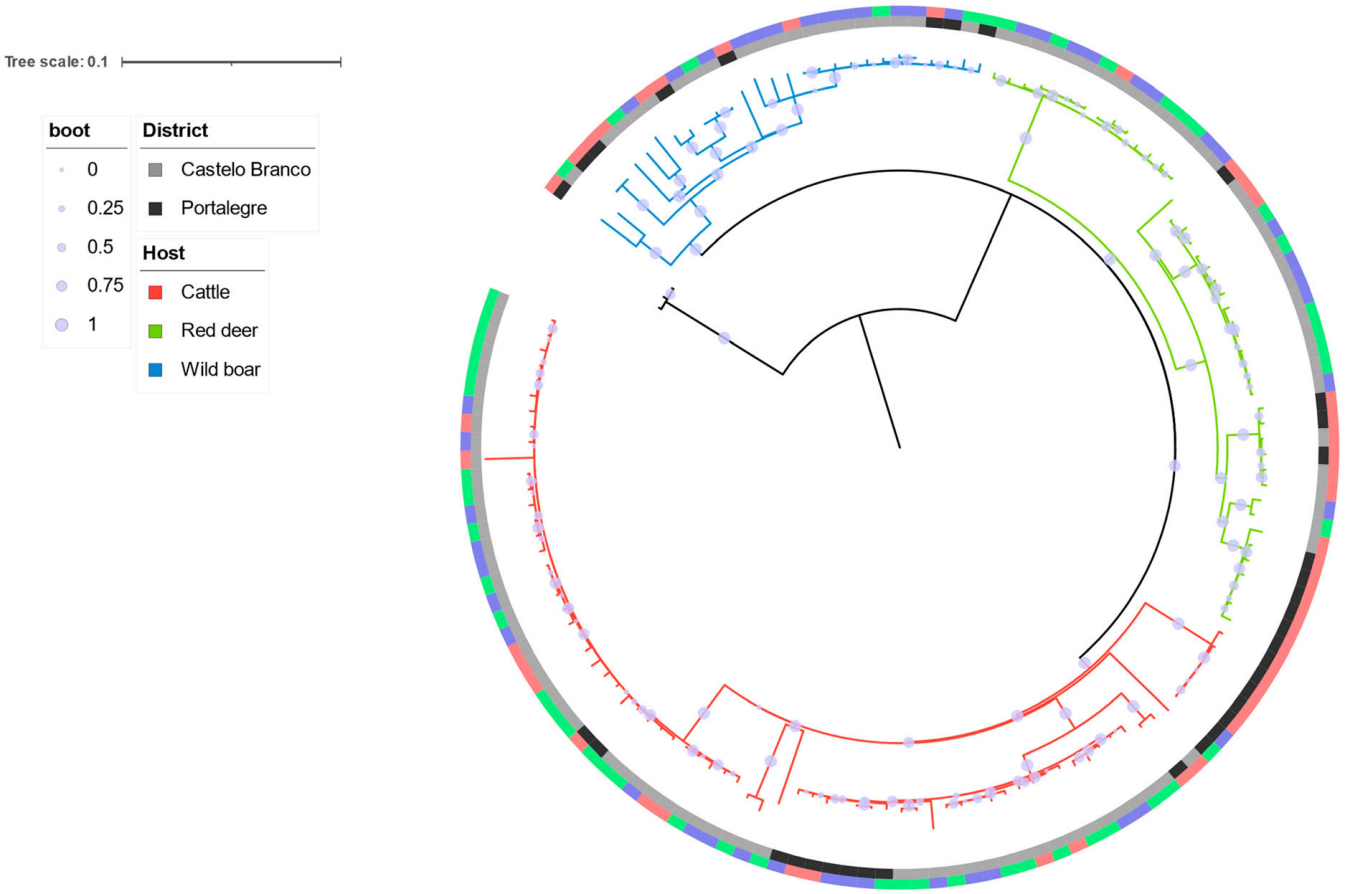
Phylogenetic analysis of 144 Eu2 *Mycobacterium bovis* from the central-east animal TB hotspot area in Portugal. A GTR (General Time Reversible) model and maximum-likelihood approach were used. Branch colours represent the three major clades. Host and district metadata are also highlighted for each genome. The scale represents the number of nucleotide substitutions. Boot – bootstrap values obtained from 1000 runs.

The phylogenetic distribution of SNPs grouped Eu2 *M. bovis* isolates from the central-eastern TB hotspot, recovered between 2002 and 2021, into three clades that evolved independently from each other (Figure 3). The so-called red clade was the largest one, accounting for 72 strains (50%), the green clade comprised 44 strains (31%), and the blue clade was the smallest, with only 28 strains (19%). All major clades included strains from all three host species and both geographic locations.

### The temporal signal of the major red clade is sufficiently informative for phylogenetic inferences of evolutionary rate and time scale

Before the Bayesian phylogenomic analysis, the sampling times of the genomes were tested for suitability to calibrate the molecular clock. The temporal signal of the Eu2 *M. bovis* population was assessed using both TempEst and LSD software. This evaluation was performed by considering either the entire population or stratifying per each phylogenetic clade. Root-to-tip analysis evidenced that the population as a whole had no temporal signal (r^2 ^= 0.06), but each of the three major clades appeared to have moderate clock-like behaviour (red clade, r^2 ^= 0.19; green clade, r^2 ^= 0.23; blue clade, r^2 ^= 0.23). Subsequently, each of these three major clades was tested individually using date-randomization analysis performed by LSD. Only the major red clade (50% of all Eu2 isolates) showed no overlap between the 95% confidence interval (CI) of the substitution rate of the original dataset and the 95% CI of the ten randomized ones (Supplementary Figure 1), thus supporting the existence of a strong temporal signal in this clade, surpassing the phylodynamic threshold. The best nucleotide substitution model for this clade was assessed using jModelTest2, with the TMP1uf model being the best-fitting one, according to BIC analysis (Supplementary Table 3).

### Phylodynamic analyses of the major Eu2 clade reveal a tMRCA in the eighteenth century and an effective reproduction number suggesting endemization

The major clade of the Eu2 clonal complex contained strains that were recovered between 2002 and 2021, from cattle (n = 19), red deer (n = 32), and wild boar (n = 21) from both districts under analysis (Castelo Branco, n = 54; Portalegre, n = 18). BEAST2 was used to perform a time-calibrated tree of these isolates, testing nine different models with three different molecular clocks and three different coalescent demographic priors. In our analysis, the relaxed exponential clock with a coalescent exponential population was the best-fitting model, according to the BF analysis and 95% HPD analysis (Supplementary Table 4). This model enables the variation in the evolutionary rate of each tree branch that occurs at the nodes independently of the branch length and estimates the population dynamics assuming an exponential growth over time.

**Figure 4.**
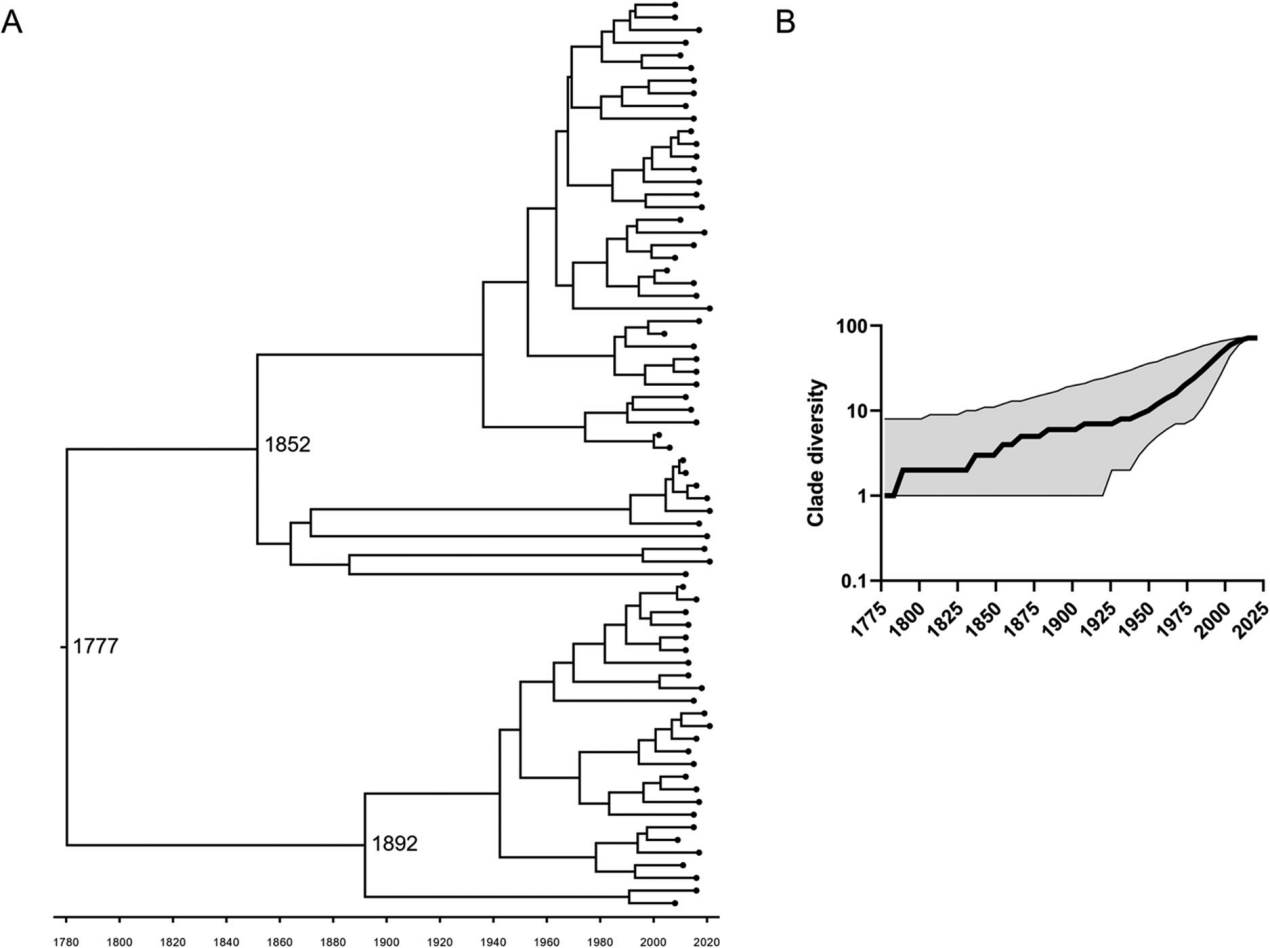
Phylodynamic analysis of the major Eu2 clade (Red clade in Figure 2, recovered between 2002 and 2021). A total of 72 genomes were analysed using BEAST2 applying a TMP1uf substitution model together with a relaxed exponential clock and a coalescent exponential population. A. Time-calibrated maximum clade credibility tree with posterior support for major nodes shown with grey bars indicating the 95% highest posterior density intervals for node date estimates. In the horizontal axis, the chronological years are represented. B. Lineage-through-time analysis using median values with a 95% confidence interval. In the horizontal axis, the chronological years are represented.

The main evolutionary parameters estimated under this Bayesian analysis represent a mean clock rate of 9.63 × 10^−5^ substitutions per-site per-year (95% highest posterior probability (HPD) [2.82 × 10^−5^–1.89 × 10^−4^]), meaning an evolutionary rate of 0.3 (95% HPD [0.1–0.6]) substitutions per genome per year, and a tMRCA estimated 244 (95% HPD [72–602]) years ago, meaning introduction around 1777 (95% HPD [1419–1949]) (Figure 4A).

A lineage through time analysis was performed to inform on clade diversification (Figure 4B). After the tMRCA, an increase in strain diversity occurred, with particular growth in the second half of the twentieth century (reaching ∼10 lineages), eventually plateauing at the beginning of the twenty-first century (reaching ∼70 lineages).

The relaxed exponential molecular clock used to describe the major clade of Eu2 is based on two major parameters, namely the effective population size at present and the growth rate, with the first being estimated to be 1291 individuals (95% HPD [379–2646]) and the second as 0.0174 new individuals/year (95% HPD [0. 0013–0. 0403]). These parameters of the exponential model enable an estimation of the effective reproduction number as long as the current number of infected individuals is known. Assuming that, at present, an average of 5809 individuals is infected in Portugal (sum of infected cattle, wild boar, and red deer individuals in the area of study, according to stochastic modelling of [38]), this leads to the rough estimation of the R_e_ as 1.01 but with a wide confidence interval (95% CI [0.14–7.25]).

### Host species transmission analyses of the major Eu2 clonal complex clade suggest the MRCA first infected the red deer population

**Figure 5.**
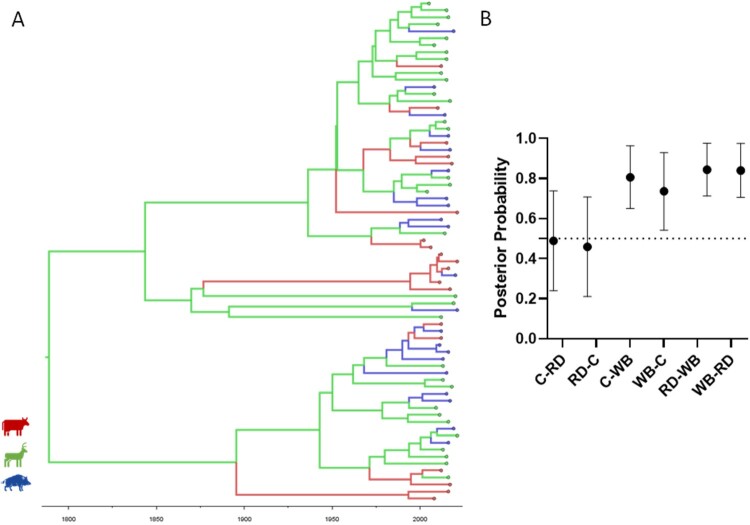
Ancestral host state reconstruction of the major European 2 clonal complex clade. A. Maximum credibility tree was estimated under a model of asymmetric host species transitions. Host species are colour-coded (cattle, red; red deer, green; wild boar, blue). B. Host state posterior probabilities under a model of asymmetric host species transition. Vertical bars represent the 95% confidence interval. The dotted line represents the posterior probability cut-off value of 0.5. Abbreviations: C, cattle; RD, red deer; WB, wild boar.

Host species transitions were evaluated using a DATM analysis approach, with the asymmetric model showing the best fitting, according to BF analysis (Supplementary Table 5). The results suggest the MRCA first infected the red deer population with subsequent host transition events toward the remaining host species (Figure 5A). Most transition events occurred intraspecifically (80%), with the majority occurring within the red deer population (56%). Moreover, based on these phylogenomic inferences and genomes dataset, endemization appears to have occurred first in the red deer population (1789) and only later in cattle (1983) and wild boar (1990) populations. Finally, transmission events were estimated as more frequent between wildlife species (wild boar-red deer, PP = 0.84) and between wild boar and cattle (PP = 0.74) (Figure 5B).

### Phylogeographic analyses of the major Eu2 clonal complex clade show that most transmission events occur inside administrative regions

Spatial diffusion of the major Eu2 clonal complex clade was evaluated through phylogeographic inferences considering administrative divisions (*i.e.* districts, at an upper administrative level, and municipalities) under both symmetric and asymmetric assumptions. Comparing all administrative division-based phylogeographic models, the symmetric coalescent model for the district as a discrete trait was the best-fitting one, according to BF analysis (Supplementary Table 5). In this model, the PP was 1.00 due to the symmetric nature of the assumptions in a two-component system, with a diffusion rate of 1.00 exchanges/year between districts.

Regarding municipality-based models, the symmetric coalescent model was the best-fitting one, inferring higher PP values between Castelo Branco – Idanha-a-Nova (PP = 1.00), Castelo de Vide – Nisa (PP = 0.89), Crato – Portalegre (PP = 0.89), and Castelo Branco – Monforte (PP = 0.60). For the municipality interaction with a high number of transitions and posterior probability support (Castelo Branco – Idanha-a-Nova), the spatial diffusion rate was 0.72 exchanges/year.

**Figure 6.**
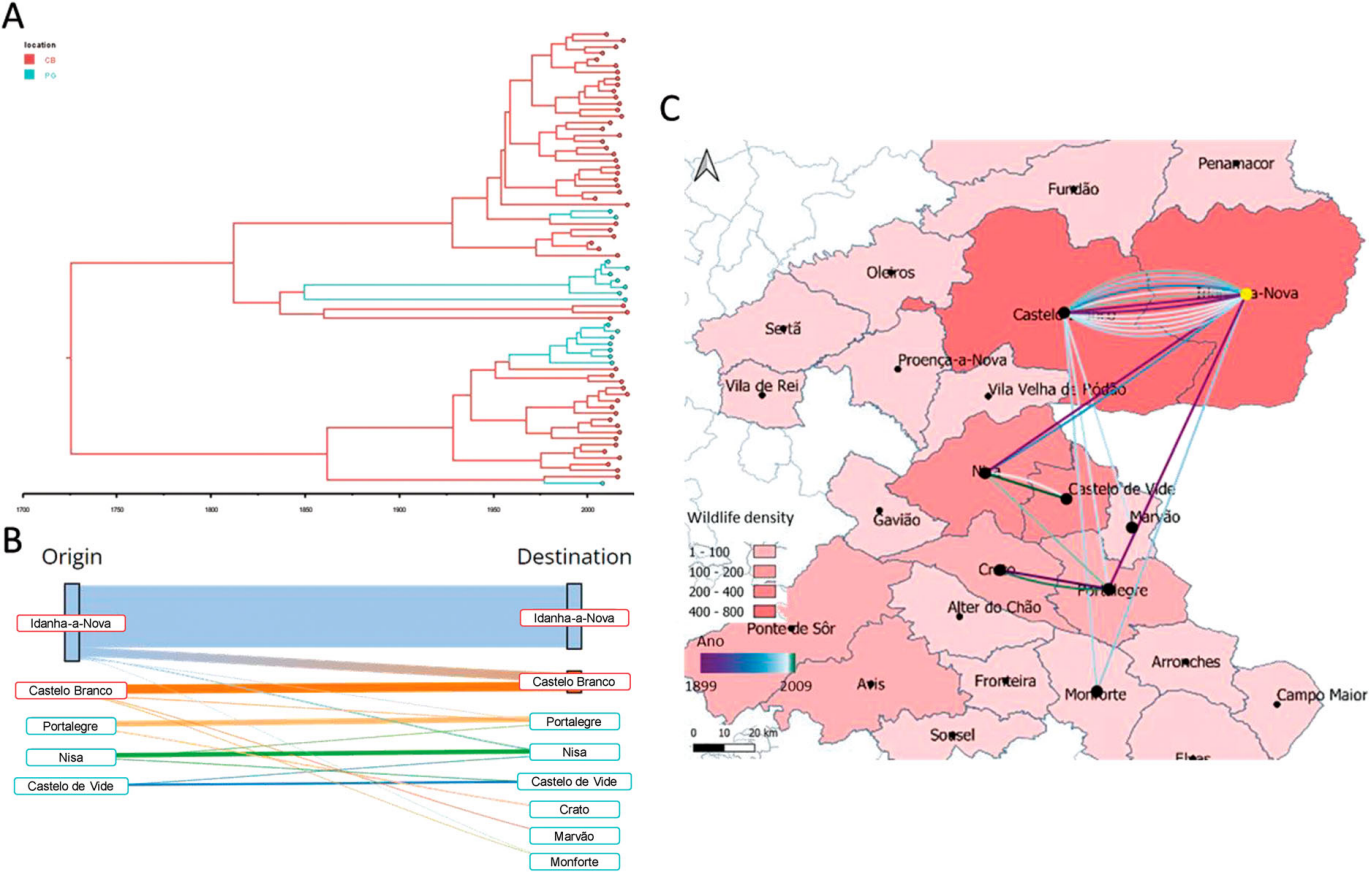
Phylogeographic analysis of the major Eu2 clade using district as a grouping factor. A. Maximum credibility tree was estimated under a model of symmetric district transitions. Districts are colour-coded (Castelo Branco, red; Portalegre, blue. B). Alluvial plot denoting both intra- and inter-municipality transitions, with transitions recovered from the internal nodes of the maximum credibility tree estimated under a symmetric municipality transition approach. C. Spatial reconstruction of inter-municipality transitions with wildlife density information per municipality (using hunting bags as proxy) and transitions colour coded by estimated year of occurrence. Transitions are positioned at the centroids of each municipality.

The data from the time-calibrated tree suggests that the MRCA-infected hosts in the Castelo Branco district (probably from Idanha-a-Nova municipality; Figure 6A and B) with subsequent geographic transitions towards the Portalegre district (Figure 6A and B). Most transition events occurred within districts (95%), particularly within municipalities (82%), with the majority of events occurring within the Idanha-a-Nova municipality (61%) (Figure 6B). Inter-municipality transitions (18%) occurred mostly between 1960 and 2000 (61.5%), with only 23% of inter-municipality transitions occurring after 2000 (Figure 6C). Before 1960, the introduction in the Portalegre district (either by the Nisa or Portalegre municipalities, according to the time-calibrated tree, Figure 6A and Supplementary Fig. 2) was first identified in 1899, while the introduction in Castelo Branco municipality (the second most represented municipality with 24% of samples) was first identified in 1904, thus highlighting the time overlap of both transitions. Note that these municipalities are the ones registering higher wildlife animal density (using hunting bags as a proxy of density).

## Discussion

The when and how are fundamental open questions in the introduction and endemization processes of *M. bovis* in the Iberian Peninsula. In particular, in Portugal, to support decision-making, there is clearly a need to dissect and date host transitions of *M. bovis* in multi-host communities. However, attempting to do this with small datasets may lead to imperfect lineage assignment and may not accurately reflect the global population structure and the underlying transmission processes. Large-scale sequencing efforts in a meaningful sampling window as the TB epidemic unfolds might help to reliably estimate epidemiological parameters, such as time to the most recent common ancestor, population growth rate, or lineage diversification rate. Here, we report the main findings from phylogenomic and phylodynamic approaches over *M. bovis* Eu2 whole-genome sequences enriched with metadata. The epidemic scenario under focus encompasses a sampling window of 19 years (up to 2021) in a recognized animal TB hotspot area in Portugal where *M. bovis* transmission is maintained at the livestock-wildlife interface [5].

In the existing *M. bovis* transmission studies supported by WGS data, authors usually discard mixed samples due to ambiguous SNP calling and the difficulty to discriminate between sequencing reads of different strains co-occurring in a given sample. Recently, a tool to overcome this limitation was developed. The SplitStrains software enables the identification and sorting of sequencing reads belonging to different strains of *Mycobacterium tuberculosis* complex bacteria occurring in the same sample [21]. Taking advantage of this tool, in our study, two cases of mixed samples were detected and the different strains present in each sample were successfully discriminated and separated. This allowed for the identification of two cases of co-infection in wild boar. The posterior phylogenomic analysis highlighted a large difference in SNPs between the major and minor strains from the same animal host, supporting the hypothesis for co-infection via two independent events instead of intra-host microevolution.

The rejection of whole-genome sequences due to the mixing of reads and ambiguous SNP calling may cause bias in transmission chain reconstruction and source tracking. Furthermore, distinguishing events of microevolution from co-infection is important to understand the different dimensions of animal TB epidemiology [17]. For example, the erroneous identification of a case as a pure infection instead of considering the microevolution or the co-infection scenarios may blur the reconstruction of epidemiological links. Microevolution detection and characterization are also important to better understand *M. bovis* biology and evolution since genetic diversification inside the host during a chronic evolution disease phenotype may represent the selection of the fittest strain for immune escape and/or horizontal transmission. These additional layers of information offered by stratification of mixed reads may thus help better define strategies for diagnosis and control.

*In silico* spoligotyping analysis revealed similar results to the previous wet lab spoligotyping analysis performed on a higher set of *M. bovis* Portuguese isolates [5], with the major difference being the predominance of SB1174 as the major spoligotype registered in the overall *M. bovis* population (i.e. all lineages), while the most predominant spoligotypes found in the Eu2 overall population were SB0122 and SB0121. Contrasting with previous results, in which SB1174 was also the most frequent spoligotype in the Portalegre district and in the wild boar population, in this study focused on Eu2 the SB0121 is predominant in Portalegre and in wild boar.

The SNP-based maximum likelihood tree discriminated three major clades, with the largest corresponding to 50% of all isolates. All major clades included strains from all host species and geographic locations (district level), suggesting common infection sources and transmission routes. This population structure is similar to the previously reported one based on a subset of isolates (n = 44), in which the Eu2 clonal complex isolates were grouped into four clusters [16]: clade A which corresponds to the Red clade in this study (41% vs 50%), clade B which corresponds to the Green clade (32% vs 31%), and Clade C and D which were clustered together in the present manuscript into a Blue clade (26% vs 19%).

To explore the evolutionary history and epidemiology of this *M. bovis* Eu2 population, we performed a temporal signal analysis which revealed a weak temporal signal when considering the entire Eu2 population, but also when considering two of the three major phylogenetic clades. A lack of temporal signal has previously been reported in this mycobacterial complex due to its inherent biological features [10, 42, 43] characterized by clonal evolution. For a slow-evolving pathogen such as *M. bovis*, the sampling window over which the genome data is collected has to be wide enough to capture sufficient sequence variation to attain the phylodynamic threshold for accurate inference. However, on the contrary, the largest phylogenetic clade (the red clade) showed a clock-like behaviour, enabling an in-depth phylodynamic analysis.

The phylodynamic analysis of this major Eu2 clade enabled the inference of important evolutionary parameters such as the tMRCA, dated from 1777, pointing to the historical transmission of Eu2 isolates in Portugal, and the substitution rate as 0.3 substitutions per-genome per-year. Previous studies focusing on Eu2 clonal complex isolates in other epidemiological scenarios showed similar substitution rates (with mean values comprised between 0.2 and 0.5) [10, 12, 14], which highlights an intrinsic substitution rate independent of the epidemiological context. These values are also similar to the ones recently inferred for the Eu3 clonal complex (between 0.4-0.6; [15]) and within the estimations obtained for *Mycobacterium tuberculosis* (between 0.04-2.2; [42]).

An advantage of applying a BEAST approach, as we did, is the estimation of other relevant epidemiological parameters, such as the effective population size at present and population growth rate. The estimation of these parameters allowed for the estimation of a third one, the effective reproduction number (R_e_), averaging at 1.01. This result is similar to other epidemiological scenarios where TB is also maintained by wildlife [44–47]. This value close to 1 possibly means a transition to endemicity, where the pathogen is stably maintained in a population through time. However, it should be noted that our estimations are circumscribed to a portion of the *M. bovis* population occurring in Portugal, i.e. ∼37.5% since the major clade subjected to phylodynamic inference is roughly half of the projected whole Eu2 population (75%). The relaxed exponential molecular clock model used to infer the effective population size of the Eu2 major clade is a projected average number based on a large time window instead of a number obtained at present. To estimate the effective reproduction number of this Eu2 clade, we assumed the current number of infected individuals projected stochastically by [38], which concerns the overall infection scenario of this multi-host community with different *M. bovis* lineages, so interpretations should be cautious.

An effective reproduction number of around one possibly reflects national efforts to detect and eliminate infected animals from the population as part of the eradication and control programme of bovine TB [7]. This effort helps decrease the number of infected animals (Φ) and increase the rate of becoming uninfectious (δ). However, due to the lack of active surveillance on wildlife animals, the majority of infected animals that are detected are those eliminated from the population through sports hunting or accidental killing [8]. This leads to higher transmission rates in these wildlife populations as previously suggested [48] since, without targeted intervention, the number of infected animals (Φ) tends to increase at a higher rate than the rate of becoming uninfectious (δ), due to the chronic evolution nature of animal TB.

The increase in abundance and expansion of both wild boar and red deer populations in the Iberian Peninsula [49, 50] attributed to the high adaptability (wild boar) and artificial management (red deer and wild boar) of these species, adds to the problem. The increase in the number and distribution of these susceptible hosts together with animal dispersion towards areas previously unaffected contribute to the spatial spread of animal TB. Moreover, animal movement and population expansion also lead to higher animal aggregation patterns. Synchronous aggregation of these sympatric wildlife species and proximity to human settlements (in the case of wild boar), facilitates the spread of mycobacteria, while asynchronous sharing of microhabitats may lead to indirect transmission via contaminated environmental matrices [1].

Transmission analysis exploring host species as a discrete trait showed that *M. bovis* transitions between hosts of the same species are common in all three species, while transmission between species occurs at a lower rate. Intraspecific transmission is more common probably due to higher direct contact rates among individuals of the same population and common space use patterns. Higher posterior probabilities of transition between wildlife species and between wild boar and cattle highlight 1) the sympatry of both red deer and wild boar, and 2) the tendency of wild boar to forage agricultural areas. The more generalistic behaviour of wild boar compared with red deer most likely contributes to disease spread by bridging individuals of different host species populations. The work of Santos et al. from the Iberian Peninsula, based on a completely different methodological approach, strongly corroborates our hypotheses. The authors also postulate that the red deer is an important driver of TB within multi-host communities, while the wild boar links the multi-host wildlife community and livestock [48].

Concomitantly, it is known that red deer show different TB lesions compared to wild boar. Red deer tend to develop more necrotic and purulent lesions, either in the respiratory or the gastrointestinal system, while wild boar usually develops necrotic, calcified lesions in the submandibular and mediastinal lymph nodes [51]. This may lead to different excretion burdens and patterns with an impact on the transmission potential of each of these host species to other hosts [52]. We may also speculate that the clade of Eu2 clonal complex under analysis in this work could be excreted in higher amounts by red deer, leading to higher within-host persistence, since red deer individuals are less likely to interact directly with both wild boars or cattle. This is because red deer prefer to inhabit forest areas in opposition to cattle which graze near human settlements. Wild boar is more habitat generalist, it tends to search more for opportunistic food sources such as those present in agroforest and pasture habitats [53, 54].

Regarding the MRCA, we found Bayesian support for the hypothesis of Eu2 introduction via the red deer population with posterior spill-over into cattle, which seems to contradict the notion of endemic TB in cattle that thus spreads to wildlife animals. However, it should be noted that our inferences are drawn from a dataset in which the number of isolates recovered from red deer is higher. Additionally, again, only a portion of the *M. bovis* population is under phylodynamic analysis, so the MRCA is restricted to the major clade.

Phylogeographic inferences indicate that there are multiple corridors for *M. bovis* transitions between hosts within the same area and between locations, possibly due to cattle movement networks and the absence of significant geographic barriers within and between administrative areas through which wildlife is free to range [55–57].

In host transmission analyses from the time-calibrated tree, the MRCA of the Eu2 clonal complex first infected red deer populations in the Castelo Branco district with subsequent *M. bovis* spread to the Portalegre district. This assumption is based on inferred transmissions through time from the calibrated tree, even though the best-fitting model is assumed to be symmetrical.

Strain differences in host tropism, pathogenesis, or host excretion routes might result in different transmission dynamics [58]. The major Eu2 clonal complex clade under analysis showed a genomic diversity stabilization in recent times. This stabilization can be the result of improved control and eradication measures, which lead to higher removal rates of infected animals, decreasing the circulation of *M. bovis*. This could also represent the random removal of minor *M. bovis* variants from circulation, reducing genomic diversity. Or perhaps there are specific Eu2 infecting genotypes that are better detected by live testing or via laboratory diagnosis.

## Conclusion

Animal TB has been a long-lasting animal health issue in Portugal, with national authorities tackling it through culling programmes focused on cattle. This focus stems from its economic importance, proximity to humans, and concerns with food safety. However, it leaves uncontrolled the other dimensions of this complex disease affecting the composition and structure of communities. In the last decade, the implementation of the first measures focusing on wildlife species maintaining animal TB in Portugal has helped to better understand the epidemiology of TB and the implications of endemicity in wildlife. Nevertheless, the surveillance system in place for wildlife species is clearly insufficient, impairing the implementation of targeted measures to reduce transmission among wildlife, and subsequently hampering the elimination of bovine TB. This has been particularly relevant in the central-east and south-east TB hotspots areas, where cattle are managed in extensive husbandry systems with increased opportunities for direct and indirect contact with wildlife species.

The data obtained in this study highlight *M. bovis* persistence in wildlife species, transmission between wild boar and cattle, and lack of transmission barriers throughout the epidemiological risk area. The body of epidemiological information provided here should be considered in the definition of new control measures, which should range from active surveillance of wildlife reservoirs, control of their density, and reduction of contacts with cattle via fencing. Among other measures, biosecurity could also be achieved through improvements to feeding and housing animals, and quarantines to limit pathogen spread.

## Supplementary Material

Supplemental Material

Supplemental Material

Supplemental Material

## Data Availability

The sequence data included in this work are deposited under Bioproject accession numbers PRJNA682618 and PRJNA946560 at a public domain server in the National Centre for Biotechnology Information (NCBI) SRA database.
