# Supplementary figures and images for: Genomic epidemiology sheds light on the emergence and spread of Mycobacterium bovis Eu2 Clonal Complex in Portugal
Source: Emerg Microbes Infect. 2023 Sep 6;12(2):2253340. doi: 10.1080/22221751.2023.2253340 (PMC10484045; doi:10.1080/22221751.2023.2253340)

location

- Castelo\_Branco
- Castelo\_Vide
- Crato
- Idanha\_Nova
- Marvão
- Monforte
- Nisa
- Portalegre

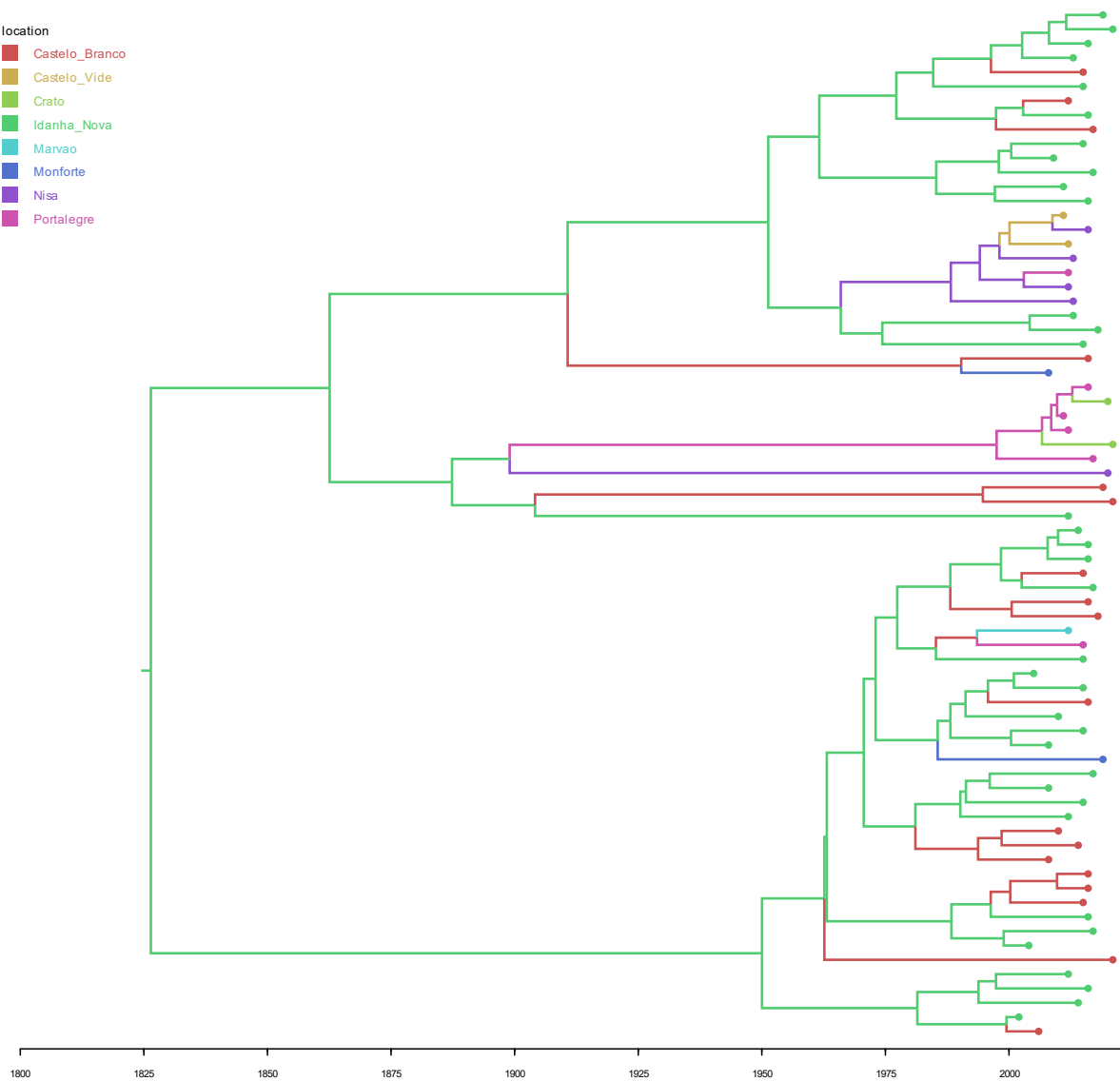

Supplement: Supplemental Material [file TEMI_A_2253340_SM7423.pdf]
