# Supplementary material for: Genomic epidemiology sheds light on the emergence and spread of Mycobacterium bovis Eu2 Clonal Complex in Portugal
Source: Emerg Microbes Infect. 2023 Sep 6;12(2):2253340. doi: 10.1080/22221751.2023.2253340 (PMC10484045; doi:10.1080/22221751.2023.2253340)
Supplement: Supplemental Material [file TEMI_A_2253340_SM4027.docx]

**Supplementary Material**

**Genomic epidemiology sheds light on the emergence and spread of *Mycobacterium bovis* Eu2 Clonal Complex in Portugal**

André C. Pereira^1,2^, Ana C. Reis^1,2^, Mónica V. Cunha^1,2*^

1. Centre for Ecology, Evolution and Environmental Changes (cE3c) & CHANGE - Global Change and Sustainability Institute, Faculdade de Ciências, Universidade de Lisboa, Lisboa, Portugal

2. Biosystems & Integrative Sciences Institute (BioISI), Faculdade de Ciências, Universidade de Lisboa, Lisboa, Portugal

*Correspondence: Mónica V. Cunha, Centre for Ecology, Evolution and Environmental Changes (cE3c), Faculdade de Ciências, Universidade de Lisboa, Campo Grande, 1749-016 Lisboa, Portugal. Email: [mscunha@ciencias.ulisboa.pt](mailto:mscunha@ciencias.ulisboa.pt)

**Keywords**: *Mycobacterium bovis*; Animal tuberculosis; Whole-genome sequencing; Phylodynamics; Phylogeography.


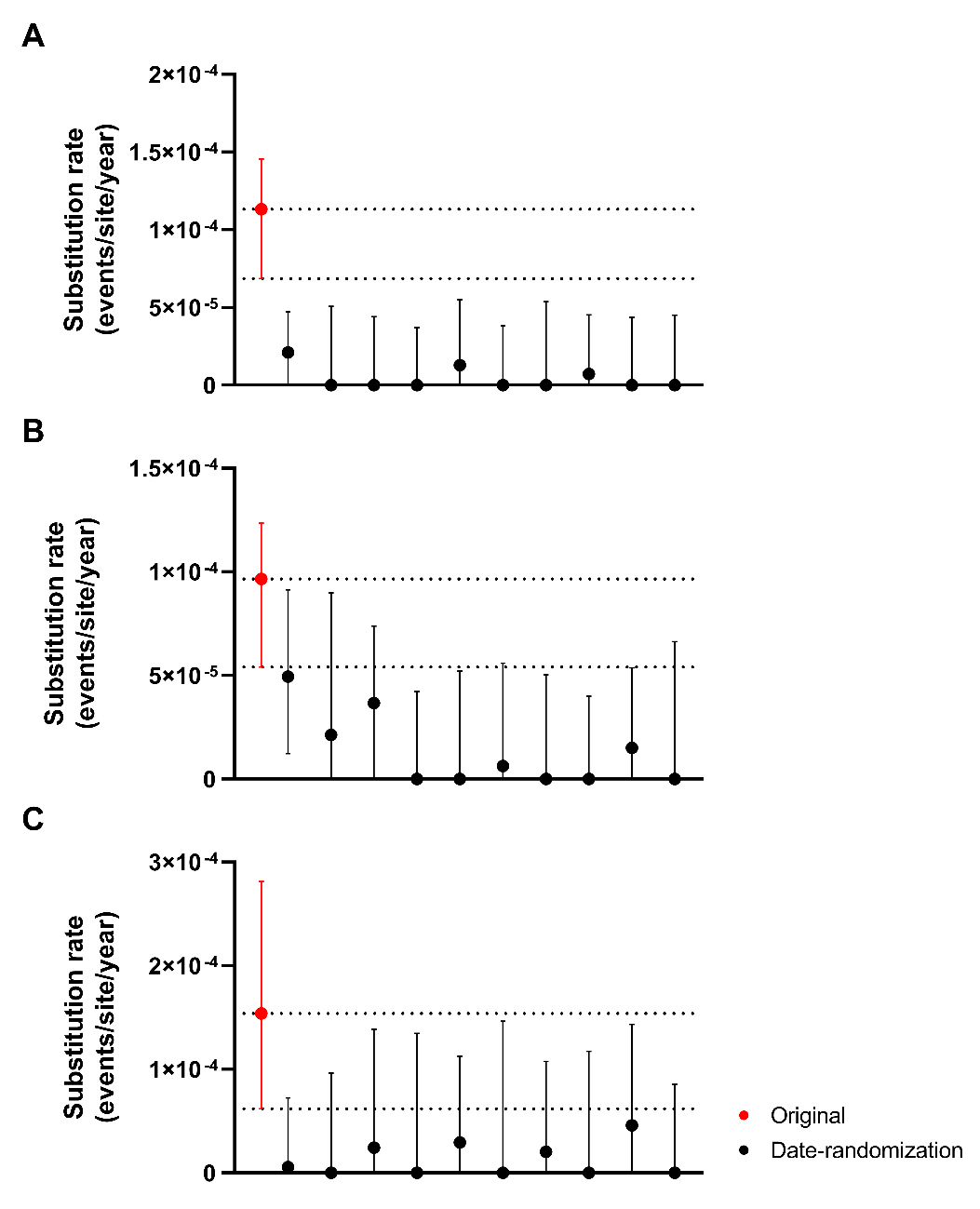


**Supplementary Figure 1** – Temporal signal analysis of European 2 clonal complex. A. Red clade. B. Green clade. C. Blue clade. Least-Squares Dating software was used to perform a date-randomization test. The original values are highlighted in red and the simulated ones are highlighted in black.

**Supplementary Figure 2** – Ancestral reconstruction of Eu2 transitions between municipalities. The maximum credibility tree was estimated under a model of symmetric transitions. Municipalities are colour-coded.
